# Clinical Outcomes of Multi‐Hole Covered Self‐Expandable Metal Stents for Malignant Distal Biliary Obstruction: A Retrospective Comparative Study With an Exploratory Evaluation of 6‐mm Devices

**DOI:** 10.1002/deo2.70411

**Published:** 2026-08-24

**Authors:** Akinobu Koiwai, Yuki Miyashita, Nana Inomata, Takehito Ito, Morihisa Hirota, Kennichi Satoh

**Affiliations:** ^1^ Division of Gastroenterology Tohoku Medical and Pharmaceutical University Miyagi Japan

**Keywords:** cholecystitis, ERCP, multi‐hole SEMS, post‐ERCP pancreatitis, RBO

## Abstract

**Objectives:**

Covered self‐expandable metal stents (CSEMS) are widely used for malignant distal biliary obstruction (MDBO), but cholecystitis, post‐endoscopic retrograde cholangiopancreatography (ERCP) pancreatitis (PEP), stent migration, and recurrent biliary obstruction (RBO) remain clinically important limitations. Multi‐hole SEMS (MHSEMS) is a novel CSEMS designed to preserve side‐branch drainage and reduce migration. We evaluated MHSEMS compared with conventional CSEMS and explored the potential impact of a newly available 6‐mm MHSEMS.

**Methods:**

This retrospective single‐center study included consecutive patients who underwent initial transpapillary SEMS placement for MDBO between September 2015 and December 2025. Study‐I compared MHSEMS versus conventional CSEMS limited to 8–10 mm diameter stents. Study‐II included MHSEMS cases only and compared 6‐mm versus 8–10 mm MHSEMS. Outcomes included cholecystitis, PEP, RBO, time to RBO (TRBO), and RBO causes.

**Results:**

Study‐I included 148 patients; median follow‐up was 80.5 versus 81.5 days (*p* = 0.761). Overall cholecystitis was 1/34 versus 23/98 (*p* = 0.008). MHSEMS was independently protective for cholecystitis (odds ratio [OR] 0.0879; 95% confidence interval [95%CI] 0.0111–0.696), whereas cystic duct involvement increased risk (OR 3.190; 95%CI 1.160–8.720). PEP was 4/36 versus 18/112 (*p* = 0.595). RBO was 1/36 versus 28/112, including migration in 0 versus 9. TRBO was longer with MHSEMS (log‐rank *p* = 0.023). Study‐II included 64 MHSEMS cases; PEP was 1/28 versus 4/36 (*p* = 0.375), RBO was 1/28 versus 1/36, and no migration occurred.

**Conclusions:**

MHSEMS was associated with fewer cholecystitis events and migration and longer TRBO versus conventional CSEMS. The potential benefit of 6‐mm MHSEMS for reducing PEP remains exploratory and requires confirmation in longer prospective studies.

## 1 Introduction

Malignant distal biliary obstruction (MDBO) frequently causes obstructive jaundice and cholangitis with deterioration of quality of life and delays in systemic therapy. Endoscopic transpapillary biliary drainage via endoscopic retrograde cholangiopancreatography (ERCP) is a standard intervention, and self‐expandable metal stents (SEMS) are often preferred over plastic stents when longer patency and fewer reinterventions are desired. Randomized trials and meta‐analyses have shown superior stent patency and reduced reintervention rates with SEMS compared with plastic stents in malignant biliary obstruction [1, 2].

Covered SEMS (CSEMS) were developed to prevent tumor ingrowth and may provide improved functional patency compared with uncovered SEMS [3]. However, recurrent biliary obstruction (RBO) and device‐ or procedure‐related adverse events (AEs) remain clinically important. Cholecystitis after SEMS placement can require urgent gallbladder drainage and may interrupt anticancer therapy, and tumor involvement of the cystic duct orifice has been repeatedly reported as a major risk factor [4, 5]. Post‐ERCP pancreatitis (PEP) is another important AE and is strongly influenced by patient and procedural risk factors such as difficult cannulation and pancreatic duct manipulation.

Multi‐hole SEMS (MHSEMS) is a modified SEMS incorporating multiple small side holes along the covering membrane. This design may preserve lateral drainage from side branches (including near the cystic duct) while maintaining the barrier effect of the covering, and may also reduce migration by improving anchoring. Recent observational studies have evaluated MHSEMS in MDBO and related malignant extrahepatic obstruction, suggesting potential antimigration advantages, while patency and AE profiles remain incompletely established [6, 7, 8]. In addition, a small‐diameter (6‐mm) MHSEMS has recently become available, raising the hypothesis that downsizing might mitigate papillary or pancreatic duct compression and reduce PEP without compromising patency, consistent with increasing interest in small‐diameter fully CSEMS strategies.

We therefore conducted a retrospective single‐center study to evaluate MHSEMS in MDBO. Study‐I compared 8–10 mm MHSEMS versus CSEMS. Study‐II explored outcomes among MHSEMS cases according to stent diameter (6 mm vs. 8–10 mm).

## 2 Methods

### 2.1 Study Design and Patients

**FIGURE 1:**
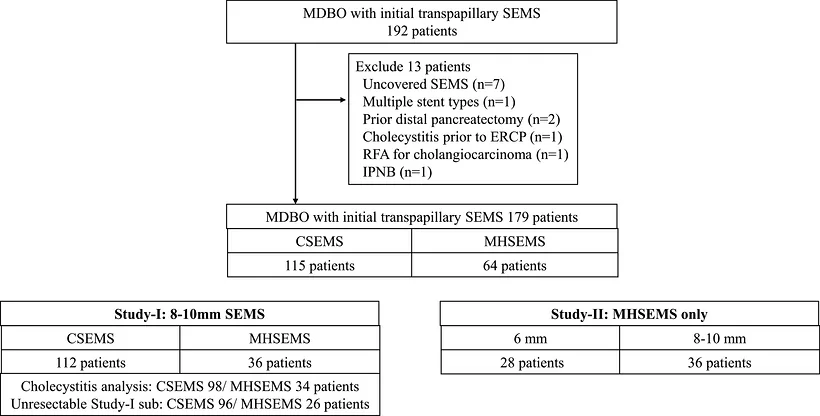
Flow diagram of patient selection and analytic cohorts. MDBO: malignant distal biliary obstruction, SEMS: self‐expandable metal stent, ERCP: endoscopic retrograde cholangiopancreatography, CSEMS: covered self‐expandable metal stent, MHSEMS: multi‐hole self‐expandable metal stent.

This retrospective, single‐center, case‐control study included consecutive patients who underwent initial transpapillary SEMS placement for MDBO between September 2015 and December 2025. During the study period, 192 patients underwent initial transpapillary biliary drainage with SEMS for MDBO, of which 13 were excluded (history of distal pancreatectomy, cholecystitis prior to ERCP, uncovered SEMS, multiple stent types at index procedure, radiofrequency ablation for cholangiocarcinoma, and intraductal papillary neoplasm of bile duct IPNB). The remaining 179 patients constituted the eligible cohort (Figure 1).

### 2.2 Endoscopic Procedures

ERCP was performed under fluoroscopic guidance. Of the 179 patients, 169 underwent pre‐procedural rectal administration of diclofenac suppositories (50 mg), given 30 min before ERCP; a reduced dose of 25 mg was administered in patients with advanced age or impaired renal function. Papillary intervention (endoscopic sphincterotomy [EST] or endoscopic papillary balloon dilation) and prophylactic pancreatic duct stenting were selected at the endoscopist's discretion. Stent length was selected according to cholangiographic assessment and stricture length. Prophylactic gallbladder stenting was not performed in any patient at the time of index ERCP/SEMS placement.

### 2.3 Type of SEMS

The MHSEMS (HANAROSTENT Biliary Multi‐hole; Boston Scientific Corporation, Marlborough, MA, USA) used in the present study is a novel type of covered SEMS to improve biliary drainage while minimizing AEs such as cholecystitis and pancreatitis. Unlike conventional CSEMS, MHSEMS incorporate a unique structure featuring multiple side holes along the stent body. This design aims to maintain the barrier function of the covering while allowing lateral drainage of bile or pancreatic secretions.

The CSEMS used in the present study were HANAROSTENT Biliary Full Cover NEO (Boston Scientific Corporation), BONA‐STENT Biliary Covered with Lasso (Medico's Hirata, Osaka, Japan), and Niti‐S ComVi Stent (Taewoong Medical, Gyeonggi, South Korea). The CSEMS group included multiple models, including fully covered and partially covered designs. Therefore, this group was defined as conventional CSEMS rather than a single uniform device. The choice of stent type was not protocolized. Device selection was determined by the attending endoscopist based on device availability, time period, and perceived risk of AEs.

### 2.4 Study Cohorts

#### 2.4.1 Study‐I (Primary Analysis)

Because 8‐mm CSEMS has been reported to be non‐inferior to 10‐mm CSEMS in MDBO regarding TRBO and AEs [9], Study‐I was restricted to 8–10 mm stents to reduce confounding by diameter. Study‐I compared MHSEMS versus CSEMS.

To address potential bias introduced by preoperative cases and subsequent surgical resection, we performed a subgroup analysis restricted to unresectable cases [Study‐I (sub)].

#### 2.4.2 Study‐II (Exploratory Analysis)

Study‐II included all MHSEMS cases and compared 6‐mm versus 8–10 mm MHSEMS to explore whether a smaller diameter might reduce PEP without compromising TRBO.

### 2.5 Definitions

Technical success was defined as successful SEMS deployment across the target stricture in the intended position. Clinical success, RBO, and TRBO were defined according to the TOKYO criteria 2024 [10]. Non‐RBO rates at 3 and 6 months were estimated from Kaplan‐Meier curves. Causes of RBO were categorized as obstruction or migration. Cholecystitis (post‐ERCP cholecystitis) was diagnosed based on clinical, laboratory, and imaging findings consistent with Tokyo Guidelines 2018 [11]. “Early cholecystitis” was defined as onset within 14 days after SEMS placement. PEP was diagnosed according to the definitions by Cotton et al. [12]. Cholecystitis analysis excluded patients with prior cholecystectomy. Non‐RBO rates at 3 and 6 months were estimated using the Kaplan‐Meier method.

### 2.6 Risk Factors

For cholecystitis, candidate predictors were sex, age, primary malignancy type, SEMS type (MHSEMS vs. CSEMS), SEMS diameter, chemotherapy, cystic duct involvement, and gallstones. For PEP, candidate predictors included sex, age, primary malignancy type, SEMS type, SEMS diameter, rectal non‐steroidal anti‐inflammatory drugs prophylaxis, EST (yes/no), initial versus non‐initial biliary drainage, main pancreatic duct (MPD) obstruction, pancreatography, prophylactic pancreatic duct stenting, and pancreatic guidewire‐assisted cannulation.

### 2.7 Statistical Analysis

Continuous variables were compared using the Mann‐Whitney U test, and categorical variables were compared using Fisher's exact test. Univariable logistic regression was used to calculate odds ratios (ORs) and 95% confidence intervals (CIs) for candidate categorical predictors. Multivariable logistic regression was performed for overall cholecystitis using variables associated with overall cholecystitis in univariable analysis. TRBO was analyzed using Kaplan‐Meier methods and compared using the log‐rank test. A *p*‐value <0.05 was considered statistically significant. All statistical analyses were performed with EZR (Saitama Medical Center, Jichi Medical University, Saitama, Japan), which is a graphical user interface for R (The R Foundation for Statistical Computing, Vienna, Austria).

## 3 Results

Patient flow is presented in Figure 1. Among 179 eligible patients, 115 received CSEMS and 64 received MHSEMS as the initial SEMS. Stent diameters were 10 mm in 115 patients, 8 mm in 33, and 6 mm in 31.

### 3.1 Study‐I (8–10 mm MHSEMS vs. CSEMS)

Study‐I included 148 patients (MHSEMS *n* = 36; CSEMS *n* = 112). Baseline characteristics are summarized in Table 1 (I), and clinical outcomes are shown in Table 2 (I). Median follow‐up duration was comparable between groups (80.5 vs. 81.5 days, *p *= 0.761). Most baseline variables were broadly comparable between groups [Table 1 (I)]. Tumor‐related MPD obstruction did not differ significantly between the MHSEMS and CSEMS groups (27/36 vs. 79/112; *p *= 0.675). Cystic duct involvement by tumor among patients with the gallbladder in situ was similar (12/34 vs. 33/98; *p *= 1.000).

**TABLE 1:** Characteristics of patients

*Characteristics of patients (Study‐I; 8–10 mm SEMS)*			
	MHSEMS (*n* = 36)	CSEMS (*n* = 112)	*p*‐Value
Male, *n* (%)	18 (50.0)	61 (54.5)	0.703
Age, years, median (range)	73 (47–98)	75 (41–96)	0.232
Primary malignancy, *n* (%)			0.771
Pancreatic cancer	29 (80.6)	90 (80.4)	
Cholangiocarcinoma	2 (5.6)	10 (8.9)	
Other	5 (13.9)	12 (10.7)	
Preoperative case			0.079
Yes	10 (27.8)	16 (14.3)	
No	26 (72.2)	96 (85.7)	
Stent diameter, *n* (%)			0.818
10 mm	29 (80.6)	86 (76.8)	
8 mm	7 (19.4)	26 (23.2)	
Rectal NSAID prophylaxis, *n* (%)			1.000
Yes	33 (91.7)	102 (91.1)	
No	3 (8.3)	10 (8.9)	
EST, *n* (%)			0.567
Yes	33 (91.7)	96 (85.7)	
No	3 (8.3)	16 (14.3)	
Initial biliary drainage, *n* (%)			0.595
Yes	32 (88.9)	94 (83.9)	
No (non‐initial)	4 (11.1)	18 (16.1)	
Chemotherapy after SEMS, *n* (%)			1.000
Yes	19 (52.8)	60 (53.6)	
No	17 (47.2)	52 (46.4)	
MPD obstruction by tumor, *n* (%)			0.675
Yes	27 (75.0)	79 (70.5)	
No	9 (25.0)	33 (29.5)	
Pancreatography performed, *n* (%)			0.336
Yes	18 (50.0)	45 (40.2)	
No	18 (50.0)	67 (59.8)	
Pancreatic GW‐assisted cannulation, *n* (%)			0.796
Yes	6 (16.7)	17 (15.2)	
No	30 (83.3)	95 (84.8)	
Prophylactic pancreatic stent, *n* (%)			0.137
Yes	5 (13.9)	6 (5.4)	
No	31 (86.1)	106 (94.6)	
Prior cholecystectomy, *n* (%)			0.359
Yes	2 (5.6)	14 (12.5)	
No	34 (94.4)	98 (87.5)	
Cystic duct involvement, *n* (%)			1.000
Yes	12 (35.3)	33 (33.7)	
No	22 (64.7)	65 (66.3)	
Duodenal invasion, *n* (%)			0.105
Yes	12 (33.3)	21 (18.8)	
No	24 (66.7)	91 (81.2)	
Gallstones, *n* (%)			0.647
Yes	7 (20.6)	26 (26.5)	
No	27 (79.4)	72 (73.5)	
***Characteristics of patients (Study‐II; MHSEMS only)***			
	**6‐mm (*n* = 28)**	**8‐10 mm (*n* = 36)**	***p*‐Value**
Male, *n* (%)	18 (64.3)	18 (50.0)	0.314
Age, years, median (range)	80 (47‐95)	73 (47‐98)	0.014
Primary malignancy, *n* (%)			0.473
Pancreatic cancer	22 (78.6)	29 (80.6)	
Cholangiocarcinoma	4 (14.3)	2 (5.6)	
Other	2 (7.1)	5 (13.9)	
Preoperative case			0.235
Yes	4 (14.3)	10 (27.8)	
No	24 (85.7)	26 (72.2)	
Rectal NSAID prophylaxis, *n* (%)			1.000
Yes	25 (89.3)	33 (91.7)	
No	3 (10.7)	3 (8.3)	
EST, *n* (%)			1.000
Yes	26 (92.9)	33 (91.7)	
No	2 (7.1)	3 (8.3)	
Initial biliary drainage, *n* (%)			0.488
Yes	23 (82.1)	32 (88.9)	
No (non‐initial)	5 (17.9)	4 (11.1)	
MPD obstruction by tumor, *n* (%)			0.775
Yes	22 (78.6)	27 (75.0)	
No	6 (21.4)	9 (25.0)	
Pancreatography performed, *n* (%)			0.314
Yes	10 (35.7)	18 (50.0)	
No	18 (64.3)	18 (50.0)	
Pancreatic GW‐assisted cannulation, *n* (%)			0.720
Yes	3 (10.7)	6 (16.7)	
No	25 (89.3)	30 (83.3)	
Prophylactic pancreatic stent, *n* (%)			1.000
Yes	3 (10.7)	5 (13.9)	
No	25 (89.3)	31 (86.1)	
Prior cholecystectomy, *n* (%)			1.000
Yes	1 (3.6)	2 (5.6)	
No	27 (96.4)	34 (94.4)	
Cystic duct involvement, *n* (%)			0.397
Yes	6 (22.2)	12 (35.3)	
No	21 (77.8)	22 (64.7)	
Duodenal invasion, *n* (%)			0.788
Yes	8 (28.6)	12 (33.3)	
No	20 (71.4)	24 (66.7)	

CSEMS: covered self‐expandable metal stent, EST: endoscopic sphincterotomy, GW: guide wire, MHSEMS: multi‐hole self‐expandable metal stent, MPD: main pancreatic duct, NSAIDs: non‐steroidal anti‐inflammatory drugs.

**TABLE 2:** Clinical outcomes and adverse events

*Clinical outcomes and adverse events (Study‐I; 8–10 mm SEMS)*			
Outcome	MHSEMS (*n* = 36)	CSEMS (*n* = 112)	*p*‐Value
Technical success, *n* (%)	36 (100)	112 (100)	—
Clinical success, *n* (%)	36 (100)	112 (100)	—
Follow‐up duration, days, median (range)	80.5 (1–580)	81.5 (1–828)	0.761
Early cholecystitis (≦14 days), *n* (%)^a^	1 (2.9)	11 (11.2)	0.186
Overall cholecystitis, *n* (%)^a^	1 (2.9)	23 (23.5)	0.008
Post‐ERCP pancreatitis, *n* (%)	4 (11.1)	18 (16.1)	0.595
RBO, *n* (%)	1 (2.8)	28 (25.0)	0.003
RBO due to obstruction	1 (2.8)	19 (17.0)	0.046
RBO due to migration	0 (0)	9 (8.0)	0.114
Time to RBO, median (95% CI), days	NA–NA	315 (259–607)	0.023
Non‐RBO rate at 3 months, % (95% CI)	100 (NA–NA)	88.5 (79.1–93.8)	—
Non‐RBO rate at 6 months, % (95% CI)	91.7 (53.9–98.8)	78.2 (65.4–86.8)	—
***Clinical outcomes and adverse events (Study‐II; MHSEMS only)***			
**Outcome**	**6 mm (*n* = 28)**	**8‐10 mm (*n* = 36)**	***p*‐Value**
Technical success, *n* (%)	28 (100)	36 (100)	—
Clinical success, *n* (%)	28 (100)	36 (100)	—
Follow‐up duration, days, median (range)	69.5 (4–231)	80.5 (1–580)	0.242
Post‐ERCP pancreatitis, *n* (%)	1 (3.6)	4 (11.1)	0.375
Overall cholecystitis, *n* (%)^b^	0 (0)	1 (2.9)	1.000
RBO, *n* (%)	1 (3.6)	1 (2.8)	1.000
RBO due to obstruction	1 (3.6)	1 (2.8)	1.000
RBO due to migration	0 (0)	0 (0)	−
Time to RBO, median (95% CI), days	NA (137–NA)	NA (NA–NA)	0.57
Non‐RBO rate at 3 months, % (95% CI)	100 (NA–NA)	100 (NA–NA)	—
Non‐RBO rate at 6 months, % (95% CI)	83.3 (27.3–97.5)	91.7 (53.9–98.8)	—

CSEMS: covered self‐expandable metal stent, ERCP: endoscopic retrograde cholangiopancreatography, MHSEMS: multi‐hole self‐expandable metal stent, NA: not available, RBO: recurrent biliary obstruction.

^a^ Cholecystitis analyses excluded patients with prior cholecystectomy; thus, denominators differ: MHSEMS *n* = 34, CSEMS *n* = 98.

^b^ Cholecystitis analyses excluded patients with prior cholecystectomy; thus, denominators differ: 6 mm *n* = 27, 8–10 mm *n* = 34.

Cholecystitis: Sixteen patients with prior cholecystectomy were excluded, leaving 132 patients for cholecystitis analyses (MHSEMS *n* = 34, CSEMS *n* = 98). Early cholecystitis occurred in 1/34 (2.9%) in the MHSEMS group and 11/98 (11.2%) in the CSEMS group (*p *= 0.186). Univariable analysis for early cholecystitis did not identify significant predictors (Table 3A). Overall cholecystitis during follow‐up occurred in 1/34 (2.9%) versus 23/98 (23.5%), respectively (*p *= 0.008). In multivariable logistic regression, MHSEMS use was independently associated with a lower risk of cholecystitis (OR 0.0879; 95% CI 0.011–0.696; *p *= 0.021), whereas cystic duct involvement by tumor independently increased the risk (OR 3.190; 95% CI 1.160–8.720; *p *= 0.024) (Table 3B).

**TABLE 3:** Risk factor analysis for cholecystitis in Study‐I.

*(A) Univariable analysis for early cholecystitis (≦14 days) (n* = *132)*			
	Cholecystitis (+) (*n* = 12)	Cholecystitis (‐) (*n* = 120)	*p*‐Value
Male, *n* (%)	7 (58.3)	65 (54.2)	1.000
Age, years, median (range)	80.5 (62–87)	74 (41–98)	0.461
Primary malignancy, *n* (%)			0.247
Pancreatic cancer	8 (66.7)	98 (81.7)	
Cholangiocarcinoma	2 (16.7)	9 (7.5)	
Other	2 (16.7)	13 (10.8)	
Stent type, *n* (%)			0.186
MHSEMS	1 (8.3)	33 (27.5)	
CSEMS	11 (91.7)	87 (72.5)	
Stent diameter, *n* (%)			0.726
10 mm	9 (75.0)	94 (78.3)	
8 mm	3 (25.0)	26 (21.7)	
Chemotherapy after SEMS, *n* (%)			0.140
Yes	4 (33.3)	68 (56.7)	
No	8 (66.7)	52 (43.3)	
Cystic duct involvement, *n* (%)			1.000
Yes	4 (33.3)	41 (34.2)	
No	8 (66.7)	79 (65.8)	
Gallstones, *n* (%)			0.726
Yes	3 (25.0)	26 (21.7)	
No	9 (75.0)	94 (78.3)	

***(B) Univariable and multivariable analysis for overall cholecystitis (n* = *132)***				
	**Cholecystitis (+) (*n* = 24)**	**Cholecystitis (‐) (*n* = 108)**	**Univariable *p*‐value**	**Multivariable** ***p*‐value/OR (95% CI)**
Male, *n* (%)	14 (58.3)	58 (53.7)	0.821	
Age, years, median (range)	75 (46–94)	74 (41–98)	0.795	
Primary malignancy, *n* (%)			0.362	
Pancreatic cancer	17 (70.8)	89 (82.4)		
Cholangiocarcinoma	3 (12.5)	8 (7.4)		
Other	4 (16.7)	11 (10.2)		
Stent type, *n* (%)			0.008	0.021/0.0879 (0.0111–0.696)
MHSEMS	1 (4.2)	33 (30.6)		
CSEMS	23 (95.8)	75 (69.4)		
Stent diameter, *n* (%)			0.786	
10 mm	18 (75.0)	85 (78.7)		
8 mm	6 (25.0)	23 (21.3)		
Chemotherapy after SEMS, *n* (%)			0.656	
Yes	12 (50.0)	60 (55.6)		
No	12 (50.0)	48 (44.4)		
Cystic duct involvement, *n* (%)			0.031	0.024/3.190 (1.160–8.720)
Yes	13 (54.2)	32 (29.6)		
No	11 (45.8)	76 (70.4)		
Gallstones, *n* (%)			0.786	
Yes	6 (25.0)	23 (21.3)		
No	18 (75.0)	85 (78.7)		

CSEMS: covered self‐expandable metal stent, MHSEMS: multi‐hole self‐expandable metal stent, OR: odds ratio.

PEP: PEP occurred in 4/36 (11.1%) in the MHSEMS group and 18/112 (16.1%) in the CSEMS group (*p *= 0.595) [Table 2 (I)]. In univariable analysis, only initial biliary drainage was associated with PEP (*p *= 0.045), whereas SEMS type and SEMS diameter, and tumor‐related MPD obstruction were not significantly associated with PEP [Table 4 (I)].

**TABLE 4:** Risk factor analyses for post‐ERCP pancreatitis (PEP).

*Study‐I (8–10 mm; n* = *148): Univariable analysis*			
	PEP (+) (*n* = 22)	PEP (‐) (*n* = 126)	*p*‐Value
Male, *n* (%)	11 (50.0)	68 (54.0)	0.818
Age, years, median (range)	75.5 (44–98)	70 (41–96)	0.052
Primary malignancy, *n* (%)			0.565
Pancreatic cancer	17 (77.3)	102 (81.0)	
Cholangiocarcinoma	3 (13.6)	9 (7.1)	
Other	2 (9.1)	15 (11.9)	
Stent type, *n* (%)			0.595
MHSEMS	4 (18.2)	32 (25.4)	
CSEMS	18 (81.8)	94 (74.6)	
Stent diameter, *n* (%)			0.784
10 mm	18 (81.8)	97 (77.0)	
8 mm	4 (18.2)	29 (23.0)	
Rectal NSAID prophylaxis, *n* (%)			1.000
Yes	20 (90.9)	115 (91.3)	
No	2 (9.1)	11 (8.7)	
EST, *n* (%)			1.000
Yes	19 (86.4)	110 (87.3)	
No	3 (13.6)	16 (12.7)	
Initial biliary drainage, *n* (%)			0.045
Yes	22 (100)	104 (82.5)	
No (non‐initial)	0 (0)	22 (17.5)	
MPD obstruction by tumor, *n* (%)			0.072
Yes	12 (54.5)	94 (74.6)	
No	10 (45.5)	32 (25.4)	
Pancreatography performed, *n* (%)			0.818
Yes	10 (45.5)	53 (42.1)	
No	12 (54.5)	73 (57.9)	
Pancreatic GW‐assisted cannulation, *n* (%)			0.340
Yes	5 (22.7)	18 (14.3)	
No	17 (77.3)	108 (85.7)	
Prophylactic pancreatic stent, *n* (%)			1.000
Yes	1 (4.5)	10 (7.9)	
No	21 (95.5)	116 (92.1)	

***Study‐II (MHSEMS only; n* = *64): Univariable analysis***			
	**PEP (+) (*n* = 5)**	**PEP (‐) (*n* = 59)**	***p*‐Value**
Male, *n* (%)	3 (60.0)	33 (55.9)	1.000
Age, years, median (range)	76 (47–98)	82 (66–88)	0.515
Primary malignancy, *n* (%)			0.462
Pancreatic cancer	4 (80.0)	47 (79.7)	
Cholangiocarcinoma	1 (20.0)	5 (8.5)	
Other	0 (0.0)	7 (11.9)	
Stent diameter, *n* (%)			0.375
8–10 mm	4 (80.0)	32 (54.2)	
6 mm	1 (20.0)	27 (45.8)	
Rectal NSAID prophylaxis, *n* (%)			1.000
Yes	5 (100)	53 (89.8)	
No	0 (0.0)	6 (10.2)	
EST, *n* (%)			1.000
Yes	5 (100)	54 (91.5)	
No	0 (0.0)	5 (8.5)	
Initial biliary drainage, *n* (%)			1.000
Yes	5 (100)	50 (84.7)	
No (non‐initial)	0 (0.0)	9 (15.3)	
MPD obstruction by tumor, *n* (%)			0.079
Yes	2 (40.0)	47 (79.7)	
No	3 (60.0)	12 (20.3)	
Pancreatography performed, *n* (%)			1.000
Yes	2 (40.0)	26 (44.1)	
No	3 (60.0)	33 (55.9)	
Pancreatic GW‐assisted cannulation, *n* (%)			0.544
Yes	1 (20.0)	8 (13.6)	
No	4 (80.0)	51 (89.4)	
Prophylactic pancreatic stent, *n* (%)			1.000
Yes	0 (0.0)	8 (13.6)	
No	5 (100)	51 (89.4)	

CSEMS: covered self‐expandable metal stent, ERCP: endoscopic retrograde cholangiopancreatography, EST: endoscopic sphincterotomy, GW: guide wire, MHSEMS: multi‐hole self‐expandable metal stent, MPD: main pancreatic duct, NSAIDs: non‐steroidal anti‐inflammatory drugs, PEP: post‐ERCP pancreatitis.

**FIGURE 2:**
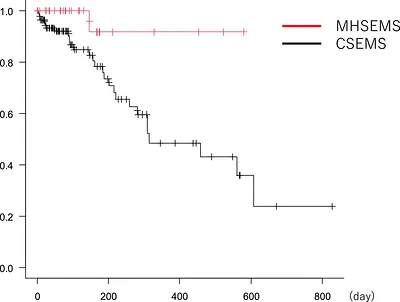
Kaplan‐Meier curves of TRBO comparing MHSEMS versus CSEMS (Study‐I). TRBO: time to recurrent biliary obstruction, MHSEMS: multi‐hole self‐expandable metal stent, CSEMS: covered self‐expandable metal stent.

RBO and TRBO: RBO occurred in 1/36 (2.8%) in the MHSEMS group and 28/112 (25.0%) in the CSEMS group (*p *= 0.003). Causes of RBO were obstruction (*n* = 1) and migration (*n* = 0) in the MHSEMS group, and obstruction (*n* = 19) and migration (*n* = 9) in the CSEMS group [Table 2 (I)]. No MHSEMS migration was observed, whereas migration occurred in nine CSEMS cases. Kaplan‐Meier analysis demonstrated significantly longer TRBO in the MHSEMS group (log‐rank *p *= 0.023) (Figure 2). Median TRBO was not reached in the MHSEMS group, whereas it was 315 days (95% CI 259–607) in the CSEMS group [Table 2 (I)].

### 3.2 Study‐I (Sub)

Study‐I (sub) included 122 patients (MHSEMS *n* = 26; CSEMS *n* = 96). In this unresectable cohort, TRBO remained significantly longer with MHSEMS (log‐rank *p *= 0.042). RBO occurred in 27/96 patients in the CSEMS group and 1/26 in the MHSEMS group (*p *= 0.008) (Table S1). Among unresectable cases with gallbladder in situ (MHSEMS *n* = 24; CSEMS *n* = 83), early cholecystitis did not differ significantly (*p *= 0.680), whereas overall cholecystitis was significantly lower with MHSEMS (*p *= 0.039) (Table S2).

### 3.3 Study‐II (MHSEMS Only)

Study‐II included 64 MHSEMS cases stratified by diameter: 6 mm (*n* = 28) and 8–10 mm (*n* = 36). Baseline characteristics are shown in Table 1 (II) and clinical outcomes are shown in Table 2 (II). Patients receiving 6‐mm MHSEMS were older (median 80 vs. 73 years; *p *= 0.014), while other procedural variables were similar. Median follow‐up duration was comparable between groups (69.5 vs. 80.5 days, *p *= 0.242).

PEP: PEP occurred in 1/28 (3.6%) in the 6‐mm group and 4/36 (11.1%) in the 8–10 mm group (*p *= 0.375). Univariable analysis did not identify significant predictors of PEP [Table 4 (II)].

**FIGURE 3:**
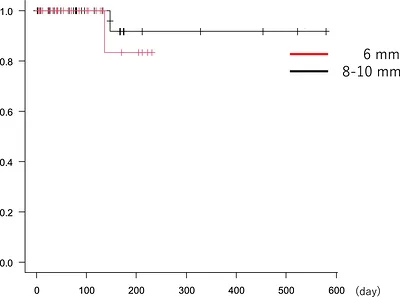
Kaplan‐Meier curves of TRBO comparing 6‐mm versus 8–10 mm MHSEMS (Study‐II). TRBO: time to recurrent biliary obstruction, MHSEMS: multi‐hole self‐expandable metal stent.

RBO, TRBO: RBO occurred in 1/28 and 1/36, respectively (*p *= 1.000); both events were due to obstruction, and no migration occurred in either group (Table 3B). TRBO did not differ significantly by diameter (log‐rank *p* = 0.57) (Figure 3).

### 3.4 Re‐intervention for RBO and Stent Exchange/ Removability

Re‐intervention for suspected RBO was performed endoscopically by ERCP. In the MHSEMS group, stent exchange was performed in seven patients, including cases undergoing elective scheduled exchange. All MHSEMS removals were technically successful without procedure‐related complications, supporting the practical feasibility of MHSEMS removal/exchange when required.

## 4 Discussion

In this single‐center retrospective study of MDBO, MHSEMS was associated with substantially fewer cholecystitis events, no observed migration, fewer RBO events, and longer TRBO compared with CSEMS in an 8–10 mm cohort. Importantly, these favorable findings persisted in analyses restricted to unresectable cases, suggesting that the observed differences were not solely driven by early surgical resection.

Cholecystitis after CSEMS placement can necessitate urgent intervention and interrupt determinant [4, 5]. In our cohort, cystic duct involvement independently increased cholecystitis risk, while MHSEMS was independently protective with a strong effect size (OR 0.0879). The overall cholecystitis incidence was markedly lower with MHSEMS, suggesting that the side‐hole design may preserve functional lateral drainage near the cystic duct region even when the covered segment overlaps this area. The lack of statistical significance for early (≤14 days) cholecystitis may reflect limited event counts, whereas the robust reduction in overall cholecystitis suggests a sustained protective effect during follow‐up. Conversely, reflux through side holes could represent a distinct mechanism of cholecystitis and warrants further evaluation in larger studies. Moreover, in unresectable patients with gallbladder in situ, overall cholecystitis remained significantly lower with MHSEMS, reinforcing the consistency of the safety.

Migration is a recognized limitation of CSEMS. In our Study‐I cohort, MHSEMS showed no migration and a markedly lower overall RBO rate, whereas migration accounted for nine of 28 RBO events in the CSEMS group. These findings support the hypothesis that MHSEMS may provide improved anchoring while maintaining the barrier effect of the covering. Prior MHSEMS studies have also highlighted low migration rates [6, 7, 13]. Consistent with fewer RBO events and no migration, TRBO was significantly longer with MHSEMS by Kaplan‐Meier analysis. To address the concern that a high proportion of preoperative cases might artificially lower RBO rates, we performed a subgroup analysis restricted to unresectable cases. In unresectable patients, TRBO remained significantly longer, and RBO remained significantly less frequent with MHSEMS, supporting that the advantage is unlikely to be explained solely by surgery‐related truncation.

PEP is multifactorial and largely driven by cannulation difficulty and pancreatic duct instrumentation [14]. In Study‐I, PEP rates were not significantly different between MHSEMS and CSEMS. In Study‐II, 6‐mm MHSEMS demonstrated a numerical reduction in PEP compared with 8–10 mm MHSEMS without evidence of inferior TRBO. Because tumor‐related MPD obstruction did not differ between the diameter groups, major confounding by this anatomic factor is less likely within Study‐II; however, the 6‐mm group was older, and event numbers were small. Therefore, the exploratory findings should be interpreted cautiously and warrant confirmation in larger cohorts. Importantly, although a formal non‐inferiority analysis for TRBO could not be performed in Study‐II due to the exploratory design and the extremely limited number of RBO events, the observed safety‐patency profile of 6‐mm MHSEMS appears biologically plausible when interpreted in the context of prior evidence on stent diameter. First, in our dataset, the PEP rate in the 6‐mm MHSEMS group (3.6%) was numerically lower than that in the 8–10 mm MHSEMS group (11.1%) and the CSEMS group in Study‐I (16.1%), suggesting a potential advantage of downsizing on PEP. However, the difference was not statistically significant; the number of events was very small. Therefore, these findings should be interpreted as exploratory and hypothesis‐generating rather than evidence that 6‐mm MHSEMS reduces PEP. Second, previous studies of conventional CSEMS suggest that reducing diameter does not necessarily compromise functional patency: an 8‐mm fully CSEMS has been reported to be non‐inferior to a 10‐mm device for TRBO in MDBO [9]. In addition, comparative data for 6‐mm versus 10‐mm fully CSEMS have reported fewer pancreatitis events with 6‐mm devices while maintaining similar overall stent function in selected cohorts [15]. Third, a key concern with small‐diameter CSEMS is migration in some clinical settings; notably, no migration was observed in our MHSEMS cohort, including 6‐mm cases. This raises the possibility that the multi‐hole structure may help retain the potential safety advantage of smaller diameter while mitigating migration‐related failure. Taken together, these considerations provide supportive context‐despite the lack of confirmatory non‐inferiority proof that 6‐mm MHSEMS may represent a promising first‐line drainage option for MDBO, provided that its safety and patency are validated in larger studies with adequate event numbers.

This study has several limitations. First, its retrospective single‐center design introduces potential selection bias and unmeasured confounding, including changes in practice over time. Second, the CSEMS group included multiple stent models with different mechanical properties and covering structures, including a partially covered design. Therefore, the comparator group was heterogeneous, which limits the validity of direct device‐to‐device comparisons. Furthermore, MHSEMS was introduced later in the study period. This non‐randomized selection was acknowledged as a potential source of selection and era bias. Third, although tumor‐related MPD obstruction was comparable between the Study‐I groups, residual confounding related to non‐randomized stent selection and unmeasured patient‐ and procedure‐related factors cannot be excluded, and propensity score‐based matched/ weighted analyses may be difficult under the constraints of the current cohort size and event numbers. Fourth, the Study‐II comparison was not designed for non‐inferiority, and the extremely limited number of RBO events precluded a reliable non‐inferiority assessment for TRBO. Accordingly, the absence of a statistically significant difference in TRBO should not be interpreted as proof of non‐inferiority. Fifth, because MHSEMS became available later in the study period, era‐related confounding cannot be excluded. In particular, changes in chemotherapy regimens, oncologic treatment strategies, imaging follow‐up, and supportive care over time may have influenced RBO, survival, and AE detection. Finally, follow‐up duration was relatively short with substantial censoring, and the number of events in the MHSEMS group – especially in Study‐II – was small. Larger multicenter studies with longer follow‐up are warranted. Such trials should prespecify a clinically acceptable non‐inferiority margin for TRBO while also evaluating potential superiority for AEs (cholecystitis and PEP) and migration.

## 5 Conclusions

In MDBO, MHSEMS was associated with a markedly lower incidence of post‐stent cholecystitis, no observed migration, fewer RBO events, and longer TRBO compared with CSEMS in an 8–10 mm cohort. In the exploratory diameter analysis within the MHSEMS cohort, 6‐mm MHSEMS showed a numerically lower incidence of PEP and no observed migration; however, because follow‐up was limited and event numbers were small, these findings should be interpreted as hypothesis‐generating. Further accumulation of cases and larger studies are required to assess long‐term patency outcomes and to determine the optimal positioning of 6‐mm MHSEMS in MDBO drainage.

## Author Contributions

**Akinobu Koiwai** conceived the study, designed the study protocol, and drafted the manuscript. **Yuki Miyashita** and **Nana Inomata** helped to develop the study measures and analysis. **Yuki Miyashita**, **Nana Inomata**, **Takehito Ito**, and **Morihisa Hirota** contributed to the case registration. **Morihisa Hirota** and **Kennichi Satoh** revised the manuscript. All authors approved the final version of the manuscript.

## Funding

The authors have nothing to report.

## Ethics Statement

The study protocol conformed to the ethical guidelines of the World Medical Association's Declaration of Helsinki and was approved by the institutional review board of Tohoku Medical and Pharmaceutical University (approval number: 2024‐2‐004‐0000).

## Consent

Informed consent was not required as this study was a retrospective analysis of existing administrative and clinical data. The patients were offered the opportunity to opt out of the study through public announcements.

## Conflicts of Interest

The authors declare no conflicts of interest.

## Supporting information




**Supporting File 1**: deo270411‐sup‐0001‐SuppMat.docx.
