# Supplementary material for: Clinical Outcomes of Multi‐Hole Covered Self‐Expandable Metal Stents for Malignant Distal Biliary Obstruction: A Retrospective Comparative Study With an Exploratory Evaluation of 6‐mm Devices
Source: DEN Open. 2026 Aug 24;7(1):e70411. doi: 10.1002/deo2.70411 (PMC13504013; doi:10.1002/deo2.70411)
Supplement: Supplementary file 1 [file DEO2-7-e70411-s001.docx]

**Supplementary Table 1**

*Subgroup analysis restricted to unresectable cases (TRBO, RBO): Study-I (sub)*

|  | **MHSEMS (n=26)** | **CSEMS (n=96)** | **P value** |
| --- | --- | --- | --- |
| TRBO, median (95% CI), days | NA (NA-NA) | 315 (221-607) | 0.042 |
| RBO, n (%) | 1 (3.8) | 27 (28.1) | 0.008 |

MHSEMS: multi-hole self-expandable metal stent, CSEMS covered self-expandable metal stent, RBO: recurrent biliary obstruction, TRBO: time to recurrent biliary obstruction, NA: not available

**Supplementary Table 2**

*Subgroup analysis restricted to unresectable cases (Cholecystitis): Study-I (sub)*

|  | **MHSEMS (n=24)** | **CSEMS (n=83)** | **P value** |
| --- | --- | --- | --- |
| Early cholecystitis (≤14 days), n (%) | 1 (4.2) | 8 (9.6) | 0.680 |
| Overall cholecystitis, n (%) | 1 (4.2) | 20 (24.1) | 0.039 |

MHSEMS: multi-hole self-expandable metal stent, CSEMS covered self-expandable metal stent
